# A two-transcript classifier model of host genes for discrimination of bacterial from viral infection in ulcerative colitis with opportunistic infections: a discovery and validation study

**DOI:** 10.3389/fimmu.2025.1642923

**Published:** 2025-09-19

**Authors:** Huipeng Zhang, Nannan Xu, Ahemala Duishanbai, Gang Huang, Jing Zhang, Guanwei Bi, Manyu Li, Gang Wang, Yanbo Yu

**Affiliations:** ^1^ Department of Gastroenterology, Qilu Hospital of Shandong University, Jinan, China; ^2^ Department of Infectious Disease, Qilu Hospital of Shandong University, Jinan, China; ^3^ Gastroenterology, Qilu Hospital (Qingdao) of Shandong University, Qingdao, China

**Keywords:** IFI44L, PI3, opportunistic infections, ulcerative colitis, bacterial infection, viral infection

## Abstract

**Aims:**

We aimed to develop and validate a classifier model to discriminate bacterial from viral infection in ulcerative colitis with opportunistic infections (UC-OI) by evaluating potential transcript signature in peripheral blood.

**Methods:**

The study comprised UC patients with bacterial or viral infection or without opportunistic infections. We screened for differentially expressed genes associated with bacterial or viral infections (*IFI44L*, *PI3* and *ITGB2*) and compared the expression levels of the genes in different infection subgroups. Subsequently, UC patients were randomly assigned (1:1) to either the discovery or validation groups. We developed a binary logistic regression model integrating the expression of candidate genes using discovery group and evaluated its discriminatory performance in validation group.

**Results:**

The expression levels of candidate genes differed significantly among infection subgroups. The *IFI44L* and *PI3* combination was the most discriminatory and was used to construct the model. The two-transcript classifier model had an AUC of 0.867 (95% CI 0.794-0.941) to discriminate bacterial and viral infections in the validation group. Its performance was better than that of PCT, CRP and ESR and was less affected by pathogen type.

**Conclusions:**

*IFI44L* and *PI3* transcript levels are robust classifiers to discriminate bacterial from viral infection in UC-OI, and measuring its levels appears to be predictive infection progression and treatment outcome in UC patients over time.

## 1 Introduction

Ulcerative colitis (UC) is a type of inflammatory bowel disease with a chronic course, prolonged duration and increasing worldwide incidence and prevalence (1, 2). In recent years, significant progress has been made in the treatment strategies for UC, and the standardization of treatment protocols has been increasing. In clinical practice, the use of drugs such as glucocorticoids, immunomodulators, and biologics has become increasingly popular, greatly improving the prognosis of patients. However, it is important to note that the above therapeutic agents, either alone or in combination, may affect the functional status of the patient’s immune system to varying degrees. With such immunomodulation, opportunistic infections may become a key safety concern for patients with UC (3–5), as they can increase the morbidity and mortality of UC and are associated with risk factors such as underlying disease, malnutrition, and intestinal surgery (6). Furthermore, the UC disease itself leads to a decline in the nutritional status of patients making them at high risk for opportunistic infections (6). Currently, although some viruses can be detected by molecular methods, and some bacterial pathogens by culture, which can be time-consuming and has low sensitivity (7, 8). Moreover, normal colonized microbes and contamination of samples can lead to false-positive results (9). Due to challenges in identifying the causative pathogen, medical practitioners often prescribe antibiotics empirically to treat UC-OI. However, misuse and overuse of antibiotics has become a severe problem, exacerbating antimicrobial resistance and Clostridium difficile superinfection (10). Differentiation between bacterial and viral infections in UC-OI is therefore crucial to avoid use of antibiotics in UC patients with non-bacterial infections.

The investigation of the host immune response constitutes an alternative approach for the identification of biomarkers that can differentiate between bacterial and viral infections in UC patients. Procalcitonin (PCT), C-reactive protein (CRP) and erythrocyte sedimentation rate (ESR) are low in sensitivity and specificity (11), but the signature of host transcripts has the potential to reveal different modes of host-pathogen interaction (12). It has been confirmed that transcriptomic biomarkers can discriminate bacterial from viral infections in acute febrile illness (13–15). Based on the gene screening in the above research results and by using the Limma package (16) to perform differential expression gene analysis on the GSE277828, GSE72849 and GSE40396 datasets in the GEO database, three potential host genes were finally screened in this study: interferon-induced protein 44-like (*IFI44L*), peptidase inhibitor 3 (*PI3*) and integrin subunit beta 2 (*ITGB2*), and attempted to investigate their value in differentiating bacterial and viral infections in UC-OI. In this study, the relative expression levels of the above candidate genes were measured by RT-PCR to investigate the differences in their expression in the healthy control (HC), the UC without opportunistic infection (UC-nonOI) group, the UC with bacterial infection (UC-B) group and the UC with viral infection (UC-V) group. By evaluating the discriminatory efficacy of each candidate gene combination, the optimal two-gene transcript combination was finally identified and used to construct and validate a two-transcript model for differentiating between bacterial and viral infections in UC patients with opportunistic infections.

## 2 Methods

### 2.1 Identification of differentially expressed genes

In this study, we focused on the expression profiles of three genes—IFI44L, PI3, and ITGB2—due to their known involvement in immune response and infection-related processes. Gene expression data were obtained from three independent GEO datasets: GSE277828, GSE72849, and GSE40396. For each dataset, differential expression analysis was performed to compare viral versus bacterial infection conditions. We calculated the log2 fold change (logFC), P-value, and adjusted P-value (adj.P.Val) for each gene and selected genes with adj.P.Val < 0.05.

Candidate gene selection followed a multi-layered approach. Initially, we identified differentially expressed genes (DEGs) from the above GEO datasets. In parallel, we searched the GeneCards database using keywords including “bacterial infection,” “viral infection,” and “immune response,” and selected genes with a Relevance score >30. These were intersected with the DEGs to highlight biologically relevant targets. To further refine the candidate pool in the context of inflammatory bowel disease (IBD), we analyzed DEGs from two IBD-specific datasets (GSE36807 and GSE75214), and performed an additional intersection. The final candidate genes were subjected to LASSO regression for feature selection, resulting in six genes of interest. Based on literature evidence (13) and classifier performance, IFI44L and PI3 were prioritized, with ITGB2 also included due to its known role in infection-related immune regulation.

### 2.2 Selection of participants and sample collection and processing

This study recruited patients who were seen or hospitalized for UC in Qilu Hospital of Shandong University, between January 2022 and May 2024, and included UC-B or UC-V or UC-nonOI. UC-B and UC-V were randomly assigned (1:1) to the discovery and validation groups by time of blood drawing via the equidistant random sampling method. Randomization was implemented via computer-generated equidistant sampling. After sorting eligible UC-B and UC-V patients according to blood collection time, a starting point of 2 was randomly selected, followed by systematic inclusion of every other patient into the discovery group. This approach ensured allocation concealment and minimized temporal bias.

Healthy medical examiners aged ≥14 years who underwent colonoscopy without evidence of intestinal disease, had no infectious disease within the last 3 months and had no comorbidities were included in the study as healthy controls (HCs). Additionally, the age and gender of HCs matched those of UC-OI. Intergroup comparability in terms of age and gender was confirmed by statistical methods (all *P* > 0.05), while expected differences were observed between UC patients and HC in terms of pathological characteristics specific to UC-OI (e.g., albumin, hemoglobin and inflammatory markers; **Supplementary Table 1**). Inclusion and exclusion criteria for UC patients are shown in **Appendix p1** and the diagnostic process for UC-B, UC-V and UC-nonOI is shown in **Supplementary Figure 1**.

The classification of acute and recovery phases was based on a combination of symptomatic, microbiological and inflammatory criteria (**Appendix p1**). Acute phase samples were collected within 72 hours of symptom onset with confirmatory pathogen evidence. Recovery phase samples were obtained 7–14 days after antimicrobial cessation, upon resolution of symptoms, negative pathogen detection and normalization of laboratory markers. Paired samples from the same patient were analyzed to control for inter-individual variability.

Two experienced specialists independently reviewed the patient’s medical records and gave the diagnosis, and the patient was included in the study only if the two specialists agreed on the diagnosis; in case of diagnostic disagreement between the two specialists, the diagnosis was arbitrated by a third specialist. This study used a double-blind design. The expert team (two gastroenterologists and one infectious disease specialist) is highly qualified and adheres to objective diagnostic standards. Three clinical experts independently diagnosed UC-OI subtypes (UC-B/UC-V) and UC-nonOI based solely on clinical/microbiological criteria without access to any transcript data or model results. Diagnoses were finalized before RNA extraction. Technicians performing RT-PCR and model analysts received only de-identified sample codes. Patient clinical information (including infection subtype) was concealed during RNA processing and model construction.

Blood samples were drawn into PAXgene Blood RNA Tubes and EDTA-anticoagulation tubes and processed. We measured expression of three candidate genes in peripheral blood by RT-PCR with the Hongshi SLAN96P PCR platform (δCt method (17); **Appendix p1-2**). PCT, CRP and ESR levels were measured as described in the **Appendix p1**. Concentrations of WBC, albumin, hemoglobin and platelet count were collected from the patient’s laboratory tests at the current inpatient or outpatient visit.

### 2.3 Disease activity assessment

We used the Mayo score (the disease activity index most commonly used in clinical practice or clinical trials) to assess disease activity of UC. The assessment protocols for the Mayo score are detailed in the **Appendix p2**.

### 2.4 Statistical analysis

In the discovery group, we expected the area under the receiver operating characteristic (ROC) curve (AUC) to be at least 0.80, based on the AUC of 0.74 of procalcitonin for diagnosis of bacterial infections. With a significance level α=0.01, we calculated a minimum required sample size of 24 UC-B and 24 UC-V, for a total of 48 patients. To ensure this minimum, we chose to include 60 patients. In the validation group, we expected the AUC to be at least 0.85, based on an AUC of 0.862 from the model in the discovery group. With a significance level α=0.01, we calculated a required sample size of 35 UC-B and 35 UC-V, for a total of 70 UC patients. To ensure this minimum, we chose to include all the remaining 92 UC patients (45 UC-B and 47 UC-V) in the study.

We performed between-group or multigroup comparisons of participant baseline characteristics and relative expression levels of candidate genes by using Fisher’s exact test (categorical variables), an independent-sample t test or a one-way analysis of variance (ANOVA) and the Benjamini-Hochberg method of *post hoc* multiple comparisons (normally distributed continuous variables), and the Mann-Whitney U test (nonnormally distributed continuous variables). We compared the expression levels of candidate genes using a paired-sample t test (normally distributed continuous variables) for dynamic changes in the acute and recovery phases of bacterial or viral infection. The risk of disease relapse was calculated using the Kaplan-Meier survival curve and Cox proportional hazards model. The risk was compared using the log-rank test. We performed Pearson’s correlation analysis to determine the correlation of Likelihood scores with Mayo scores. A two-tailed *P* < 0.05 was defined as statistically significant.

Through logistic regression analysis, we identified the most robust two-gene combination that distinguish between bacterial and viral infections in UC-OI, and constructed a logistic regression model. The classifier model was constructed in the discovery group via binary logistic regression, and the threshold for classification was derived via the Youden method. To further evaluate the robustness of the model and minimize the risk of overfitting, we performed 200 repetitions of 10-fold cross-validation on the discovery group. In each iteration, the dataset was randomly partitioned into 10 subsets, with 9 subsets used for training and the remaining one for validation. This procedure was repeated 200 times to ensure that each sample was used for both training and validation across different folds, thereby providing a stable estimate of the model’s generalizability under various data splits. In addition, age and sex were incorporated as covariates in the model. These demographic factors were forced into the model regardless of baseline statistical significance, based on their well-established clinical relevance and potential to confound host immune transcriptional responses.

With the diagnostic results from the gold standard (culture and PCR) as the reference, we performed ROC curve analysis to evaluate the performance of our two-transcript classifier model for discriminating bacterial from viral infection in UC-OI in the validation groups. We also applied ROC to compare the performance of the classifier to that of the traditional biomarkers ESR, CRP and PCT in discriminating between bacterial and viral infection. We calculated the sensitivity, specificity, positive predictive value, negative predictive value and diagnostic accuracy. 95% CIs were assessed by the Wilson score algorithm (18). The difference in AUC between pairs of ROC curves was evaluated with DeLong’s test (19).

We merged UC-V and UC-nonOI into a single group (non-bacterial) to evaluate the model in distinguishing bacterial infection from non-bacterial diseases. We also did subgroup analysis to explore whether the performance of the two-transcript classifier model was affected by pathogen subtypes (Epstein-barr virus [EBV] and Non-EBV infection).

All the statistical analyses were performed with SPSS (version 29.0.1.0), GraphPad Prism (version 10.2.0), and MedCalc (version 20.0).

## 3 Results

### 3.1 Identification of differentially expressed genes

The data extracted from the three GEO datasets for the genes IFI44L, PI3, and ITGB2 revealed the following expression profiles: IFI44L exhibited a range of logFC from -1.72 to -4.10 values across datasets, with three datasets showing statistically significant downregulation. PI3 showed significant changes in virus infection, with a logFC from 2.34 to 2.48 in three datasets, indicating upregulation, and the adjusted P-values were all smaller than 0.05, reflecting its strong robustness. ITGB2 exhibited mixed expression patterns with minimal changes from -0.28 to 0.32, while its expression pattern in GSE40396 indicated a significant alteration in some conditions. DEGs Analysis of these genes was detailed in **Table 1**.The volcano plot, DEG analysis results, LASSO cross-validation plot, and LASSO regression coefficients are provided in **Supplementary Table 5** and **Supplementary Figure 2**.

**Table 1:** Differential expressed genes analysis of *IFI44L*, *PI3* and *ITGB2* in bacterial and viral infections.

Database	*IFI44L*			*PI3*			*ITGB2*		
	logFC	*P*.val	adj.*P*.val	logFC	*P*.val	adj.*P*.val	logFC	*P*.val	adj.*P*.val
GSE277849	-4.10	8.62E-12	8.91E-09	2.36	1.78E-05	1.34E-03	-0.28	4.02E-01	4.65E-01
GSE72829	-3.21	1.67E-34	4.10E-30	2.34	6.22E-19	3.73E-16	0.12	1.81E-01	4.56E-01
GSE40396	-1.72	5.70E-02	2.24E-01	2.48	1.86E-05	2.80E-03	0.32	5.84E-05	5.07E-03

LogFC, log_2_ (fold change).

### 3.2 Description of the study participants

**Figure 1:**
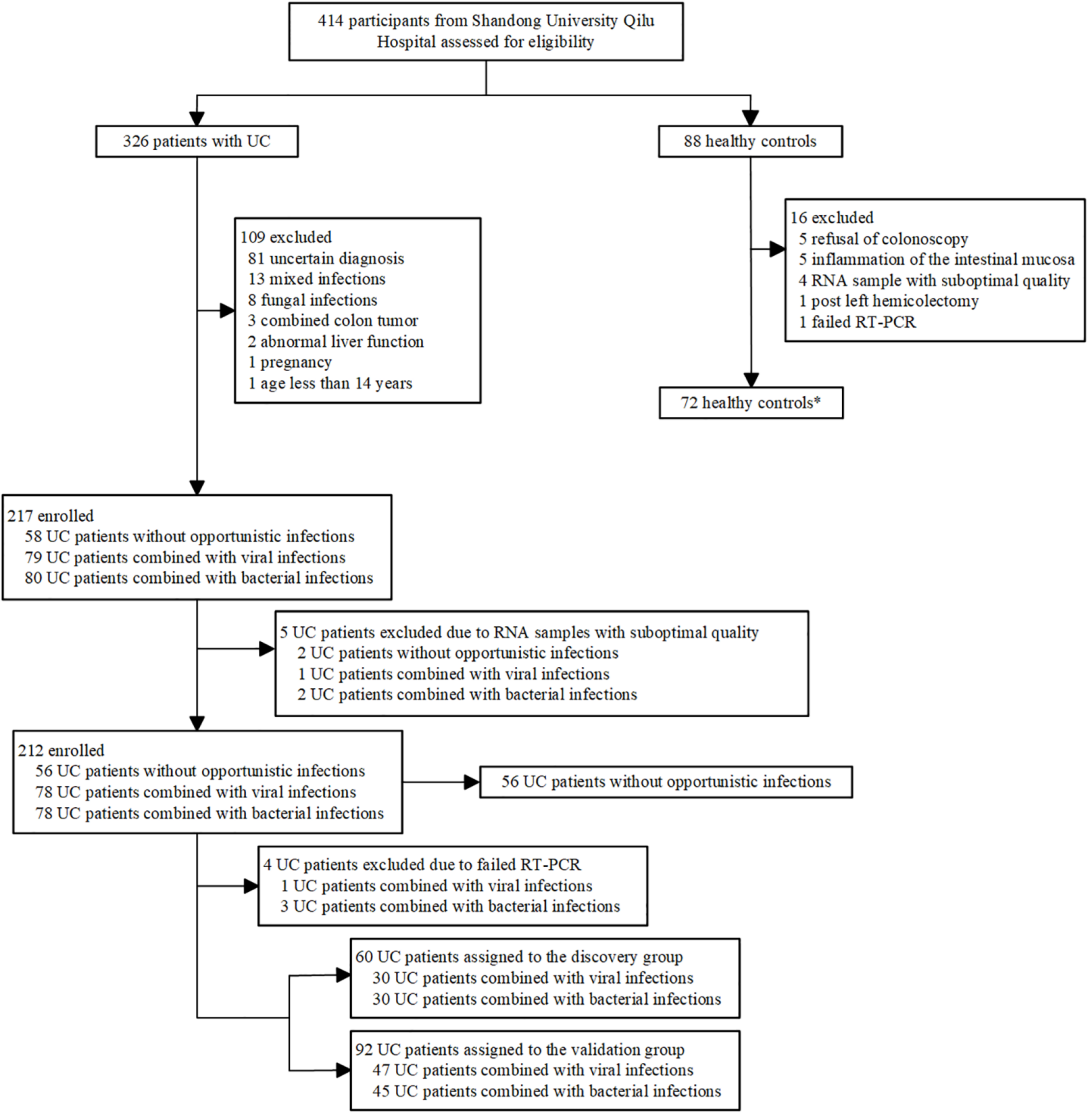
Study profile. ^*^Data from healthy controls were not used in identification or validation of gene expression signatures and were used only for comparison.

We assessed 414 participants (326 patients with UC and 88 healthy controls) for inclusion in the study. 118 patients with UC were excluded and 16 HCs were excluded (**Figure 1**). Ultimately, a total of 208 UC patients (56 UC-nonOI, 77 UC-V and 75 UC-B) and 72 HCs were included. Data from 72 HCs were used only to compare their gene expression to patients with UC. A total of 349 blood samples were successfully analyzed by RT-PCR (69 patients with UC provided two blood samples: one each from the acute phase and the recovery phase of a bacterial or viral infection). For patients with UC who provided two samples, the sample with opportunistic infections was evaluated in subsequent analyses, whereas the second sample was used only for before-and-after comparisons between the patients themselves. Further details regarding the characteristics of the study participants are included in **Supplementary Table 1**.

### 3.3 Differences in the peripheral blood relative expression levels of candidate genes

**Figure 2:**
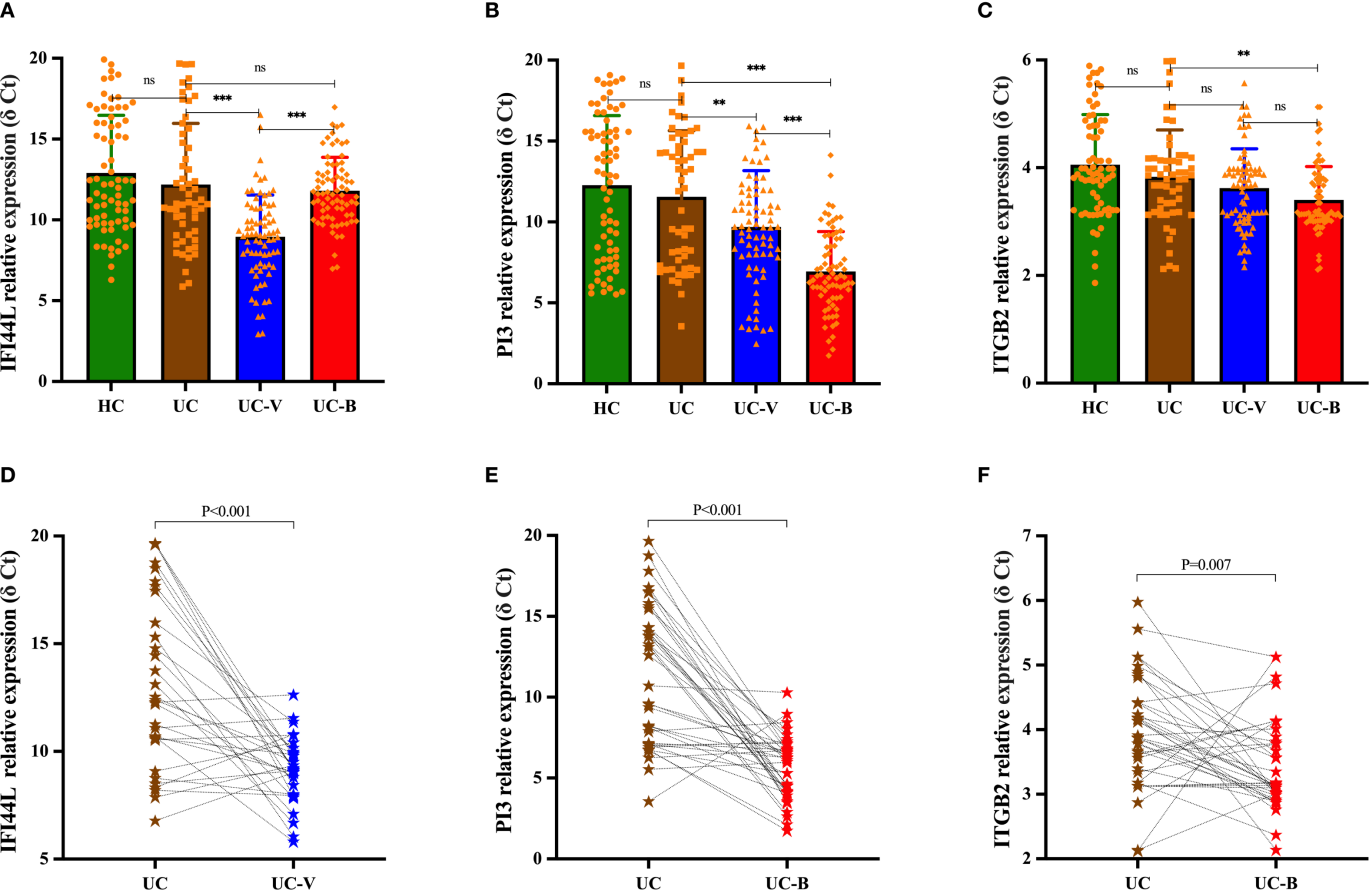
Differences in relative expression levels of candidate genes among study participants in four subgroups [**(A)** *IFI44L*; **(B)** *PI3*; **(C)** *ITGB2*]. Statistical differences were assessed using one-way analysis of variance (ANOVA); *post hoc* comparisons were made using the Benjamini-Hochberg method. Dynamics of candidate gene expression during the acute and recovery phase in UC patients with opportunistic infections [**(D)**, *IFI44L*; **(E)**, *PI3*; **(F)**, *ITGB2*]. A paired-sample t test was used to evaluate statistical differences. The relative expression level of candidate genes was expressed as δ Ct values, and higher δ Ct values indicate lower mRNA expression levels. Each graph represents an individual sample. **p < 0.01, ***p < 0.001; ns, *p* > 0.05. UC-V, UC with viral infections; UC-B, UC with bacterial infections; UC, UC without opportunistic infections (or recovery phase of opportunistic infections); HCs, healthy controls.

The relative expression levels of *IFI44L* (F = 24.381, *P* < 0.001), *PI3* (F = 31.120, *P* < 0.001), and *ITGB2* (F = 9.155, *P* < 0.001) were statistically different in the four groups of HC, UC-nonOI, UC-B and UC-V. We then used the Benjamini-Hochberg method for *post hoc* comparisons. The specific results are included in **Figures 2A–C** and **Supplementary Table 2**.

For the same UC patients, the relative expression level of *IFI44L* was increased in peripheral blood during the acute phase of viral infection compared to the recovery phase (*P* < 0.001), whereas its expression was not statistically different between the acute and recovery phases of bacterial infection (*P* = 0.197); the relative expression level of *PI3* and *ITGB2* was increased in peripheral blood during the acute phase of bacterial infection compared to the recovery phase (*PI3*, *P* < 0.001; *ITGB2*, *P* = 0.007), while there was no statistically significant difference in their expression during the acute and recovery phases of viral infection (*PI3*, *P* = 0.412; *ITGB2*, *P* = 0.605) (**Figures 2D–F**).

### 3.4 Construction and validation of the two-transcript classifier model

Thirty UC-B and 30 UC-V were assigned to the discovery group according to sampling time through the equidistant random sampling method. The remaining 92 UC patients were assigned to the validation group (45 UC-B and 47 UC-V).

In the discovery group, the results of logistic regression showed that confounders such as age (*P* = 0.284) and gender (*P* = 0.693) were unrelated to the outcome variable and the cross-validation results demonstrated consistent model performance. Among the predictors, the likelihood score was significantly associated with the outcome variable (β=5.76, SE = 1.11, z=5.18, *P* < 0.001), suggesting that higher scores markedly increase the probability of the event. In contrast, sex (β=0.13, SE = 0.59, *P* = 0.82) and age (β=0.03, SE = 0.02, *P* = 0.17) were not statistically significant, indicating that these variables had little to no impact on the outcome in this model. These results highlight that the likelihood score is the most important predictor in the model, whereas sex and age do not contribute significantly. Across 200 repetitions of 10-fold cross-validation, the mean accuracy was 0.797, the F1-score was 0.795, the precision was 0.797, the recall was 0.825, and the AUC of ROC was 0.868 (**Supplementary Figure 3**). These findings indicate that the model maintained reliable discriminative ability and stability across different training–validation partitions.

**Figure 3:**
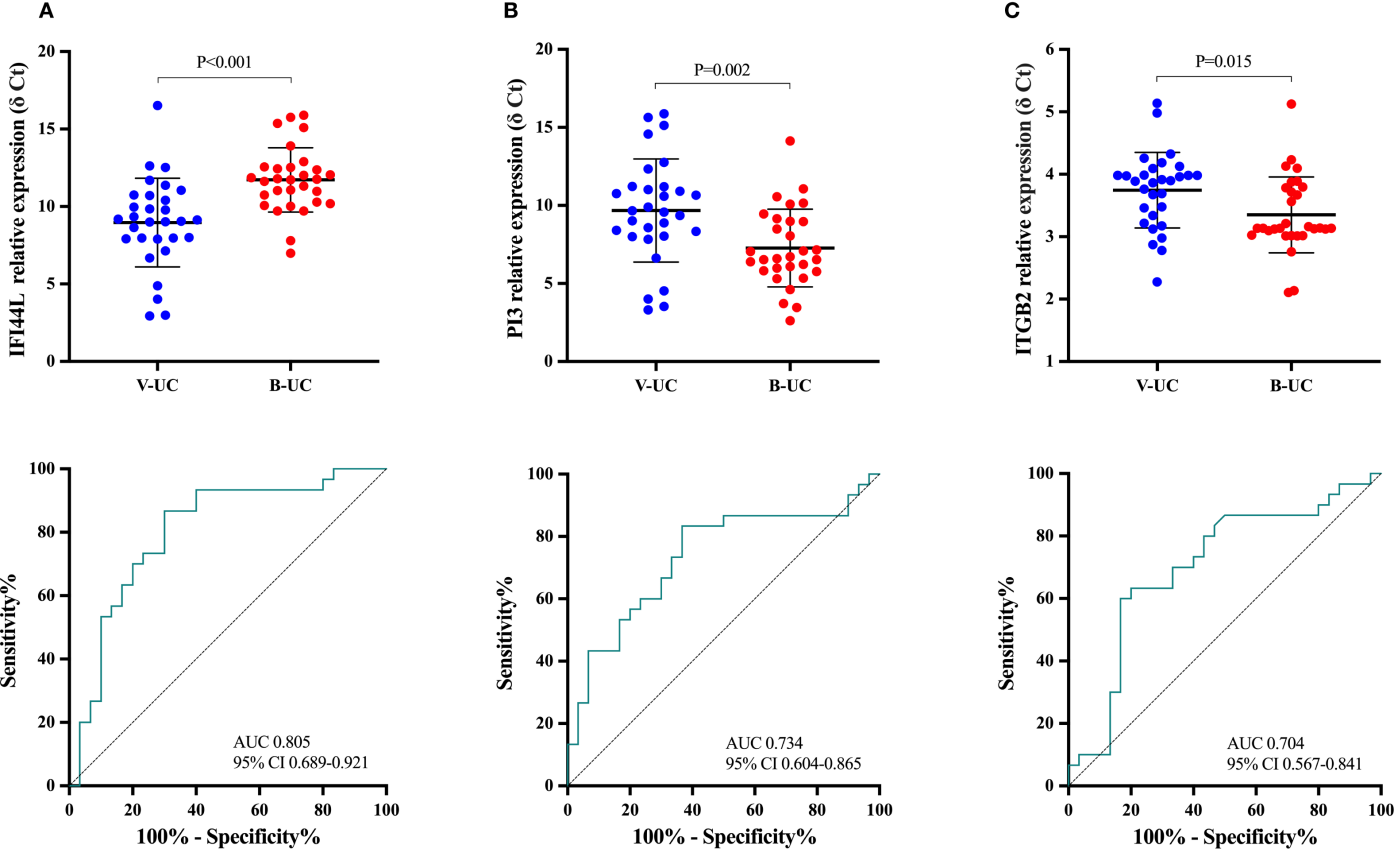
The means of relative expression of candidate genes and receiver operating characteristic curves in the discovery group. Each dot represents an individual sample. An independent-sample t test was used to evaluate statistical differences. The relative expression level of candidate genes was expressed as δ Ct values, and higher δ Ct values indicate lower mRNA expression levels. **(A)**, *IFI44L*; **(B)**, *PI3*; **(C)**: *ITGB2.* UC-V, UC with viral infections; UC-B, UC with bacterial infections.

In the 60 UC patients in the discovery group, the trends in expression of *IFI44L* and *PI3* were opposite in bacterial and viral infection, and expression differed between the two types of infection more than expression of *ITGB2* (*IFI44L*, *P* < 0.001; *PI3*, *P* = 0.002; *ITGB2*, *P* = 0.015; *IFI44L* AUC 0.805, 95% CI 0.689-0.921; *PI3* 0.734, 0.604-0.865; *ITGB2* 0.704, 0.567-0.841; **Figure 3**). Dichotomy logistic regression analysis revealed that *IFI44L* and *PI3* were the most robust combinations (AUC 0.862, 95% CI 0.760-0.964; **Supplementary Table 3**). Furthermore, *IFI44* and *PI3* remained significantly different between UC patients with bacterial and viral infections in multivariable analysis (**Supplementary Table 4**). Therefore, *IFI44L* and *PI3* were chosen as classifier genes to construct a diagnostic model.

Calculating the likelihood score of UC-B according to the relative levels of the two transcripts (*IFI44L* and *PI3*) in the discovery group using a logistic regression algorithm identified the optimal cutoff score as 0.5552794; the larger the value, the higher the likelihood of UC-B.

**Figure 4:**
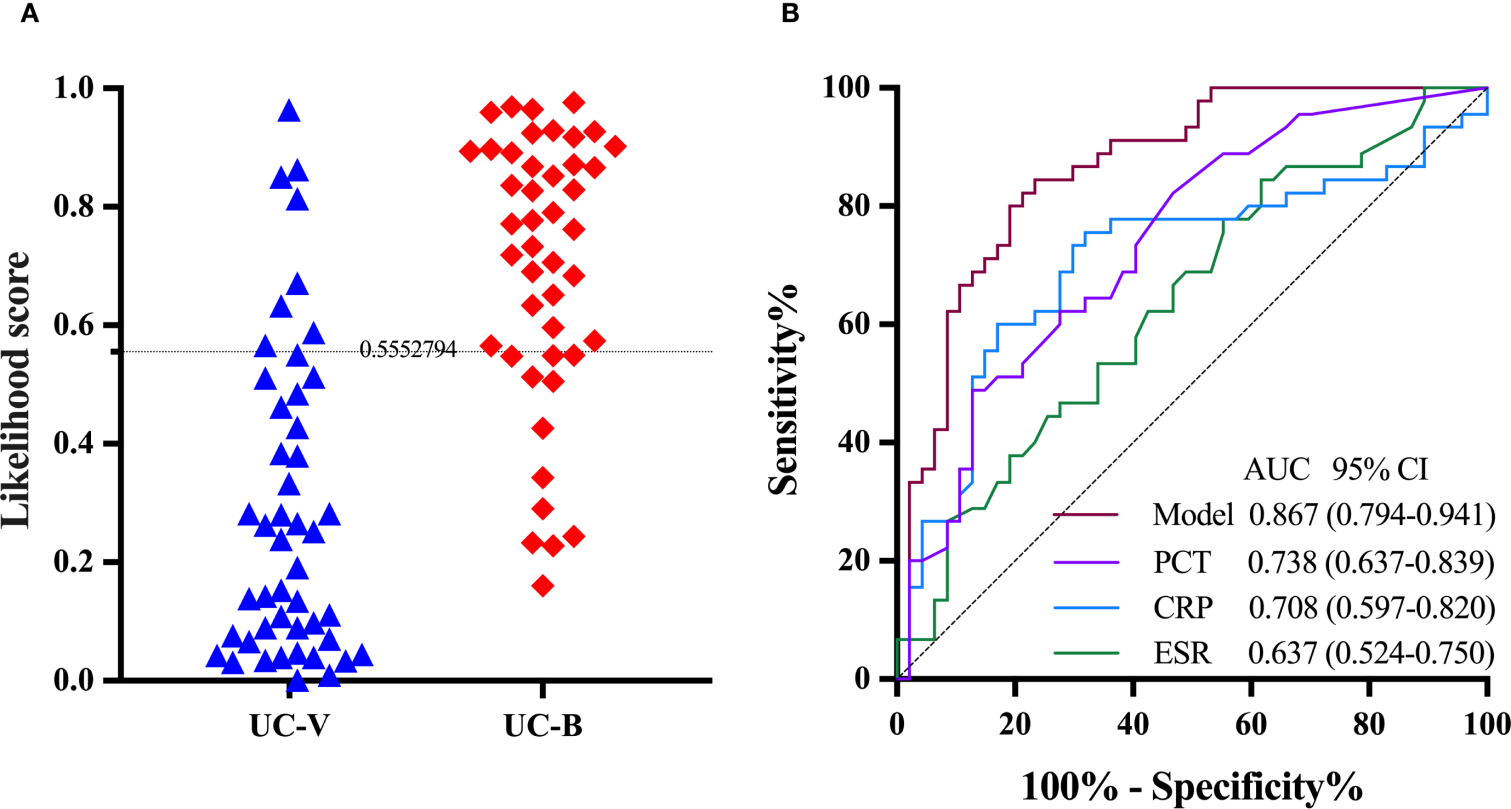
The performance of the two-transcript model to distinguish UC with bacterial infection from viral infection in the validation group. **(A)** The grey dashed line denotes the likelihood score value of 0.5552794, above which the UC with bacterial infections is favored. **(B)** PCT, CRP and ESR are included for comparison. The DeLong test was used to assess statistical differences between paired AUCs. AUC, area under the receiver operating characteristic curve; UC-V, UC with viral infections; UC-B, UC with bacterial infections; PCT, Procalcitonin; CRP, C-reactive protein; ESR, erythrocyte sedimentation rate.

Among 92 UC patients in the validation group, 41 (44.6%) had likelihood scores above the threshold (0.5552794), of whom 33 (80.5%) had bacterial infection and eight (19.5%) had viral infection. The remaining 51 (55.4%) UC patients had likelihood scores below the threshold, of whom 12 (23.5%) had bacterial infection and 39 (76.5%) had viral infection. The two-transcript classifier model in distinguishing UC with bacterial from viral infection had an AUC of 0.867 (95% CI 0.794-0.941), sensitivity of 0.733 (0.590-0.840), specificity of 0.830 (0.699-0.911), positive predictive value of 0.805 (0.660-0.898), negative predictive value of 0.765 (0.632-0.860) and diagnostic accuracy of 0.783 (0.688-0.855; **Figure 4A**). The AUC of the model was significantly higher than that of PCT (0.738, 95% CI 0.637-0.839; *P* = 0.042), CRP (0.708, 0.597-0.820; *P* = 0.019) and ESR (0.637, 0.524-0.750; *P* < 0.001; **Figure 4B**).

### 3.5 Performance of the two-transcript model to distinguish UC with bacterial from non-bacterial infections

**Figure 5:**
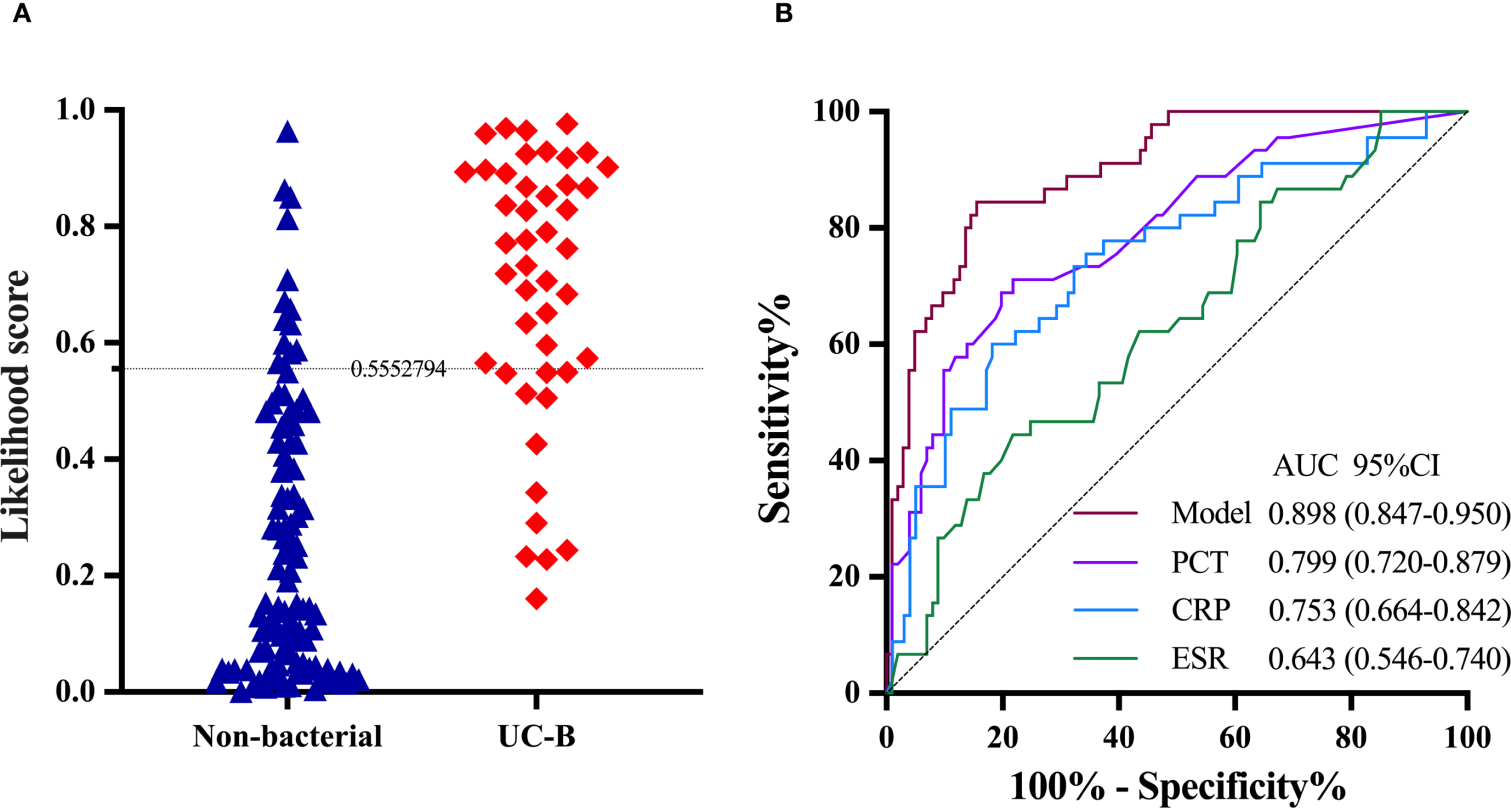
The performance of the two-transcript model to distinguish UC with bacterial infection from non-bacterial infection. **(A)** The grey dashed line denotes the likelihood score value of 0.5552794, above which the UC with bacterial infections is favored. **(B)** PCT, CRP and ESR are included for comparison. The DeLong test was used to assess statistical differences between paired AUCs. AUC, area under the receiver operating characteristic curve; UC-B, UC with bacterial infections; PCT, Procalcitonin; CRP, C-reactive protein; ESR, erythrocyte sedimentation rate.

The expression of *IFI44L* and *PI3* transcripts was significantly different in UC-nonOI and in UC-B or UC-V. We merged UC-B in the validation group and UC-nonOI into a single group (non-bacterial). Among 148 UC patients, 46 (31.1%) had likelihood scores above the threshold (0.5552794), of whom 33 (71.7%) had bacterial infections and 13 (28.3%) had viral infections or UC-nonOI. The remaining 102 (68.9%) UC patients had likelihood scores below the threshold, of whom 12 (11.8%) had bacterial infections and 90 (88.2%) had viral infections or UC-nonOI. The AUC of the model in distinguishing UC with bacterial from non-bacterial infections was 0.898 (95% CI 0.847-0.950; **Figure 5A**). The AUC of the model was significantly higher than AUCs for PCT (0.799, 95% CI 0.720-0.879; *P* = 0.028), CRP (0.753, 0.664-0.842; *P* = 0.005) and ESR (0.643, 0.546-0.740; *P* < 0.001; **Figure 5B**).

### 3.6 Performance of the two-transcript model to distinguish UC-OI from UC-nonOI

To validate the performance of model in distinguishing between UC-OI and UC-nonOI, we merged UC-B and UC-V in the validation group into a single group (UC-OI). Among 148 UC patients, 56 (%) had likelihood scores above the threshold (0.5552794), of whom 51 (91.1%) was UC-OI and six (8.9%) was UC-nonOI. The remaining 92 (%) UC patients had likelihood scores below the threshold, of whom 41 (44.6%) was UC-OI and 51 (55.4%) was UC-nonOI. The AUC of the model in distinguishing UC with bacterial from non-bacterial infections was 0.766 (95% CI 0.689-0.844; **Supplementary Figure 4**). The AUC of the model was significantly higher than AUC for ESR (0.582, 0.483-0.680, *P* = 0.001), yet lower than AUCs for PCT (0.953, 0.915-0.992; *P* < 0.001) and CRP(0.825, 0.751-0.899; *P*>0.05).

### 3.7 Assessing the effect of pathogen type on the performance of the two-transcript model

In the 22 UC patients with EBV infection, 17 (77.3%) had a likelihood score below the threshold (0.5552794) and 5 (22.7%) had a likelihood score above the threshold. The AUC of the two-transcript classifier for distinguishing EBV infection from bacterial infection was 0.815 (95% CI 0.700-0.930; **Supplementary Figure 5A**). In the 25 UC patients with non-EBV infections, 22 (88.0%) had likelihood scores below the threshold and 3 (12.0%) had likelihood scores above the threshold. The AUC for distinguishing Non-EBV infection from bacterial infection was 0.959 (0.918-1.000; **Supplementary Figure 5B**).

### 3.8 Clinical outcomes according to the two-transcript model classification

**Figure 6:**
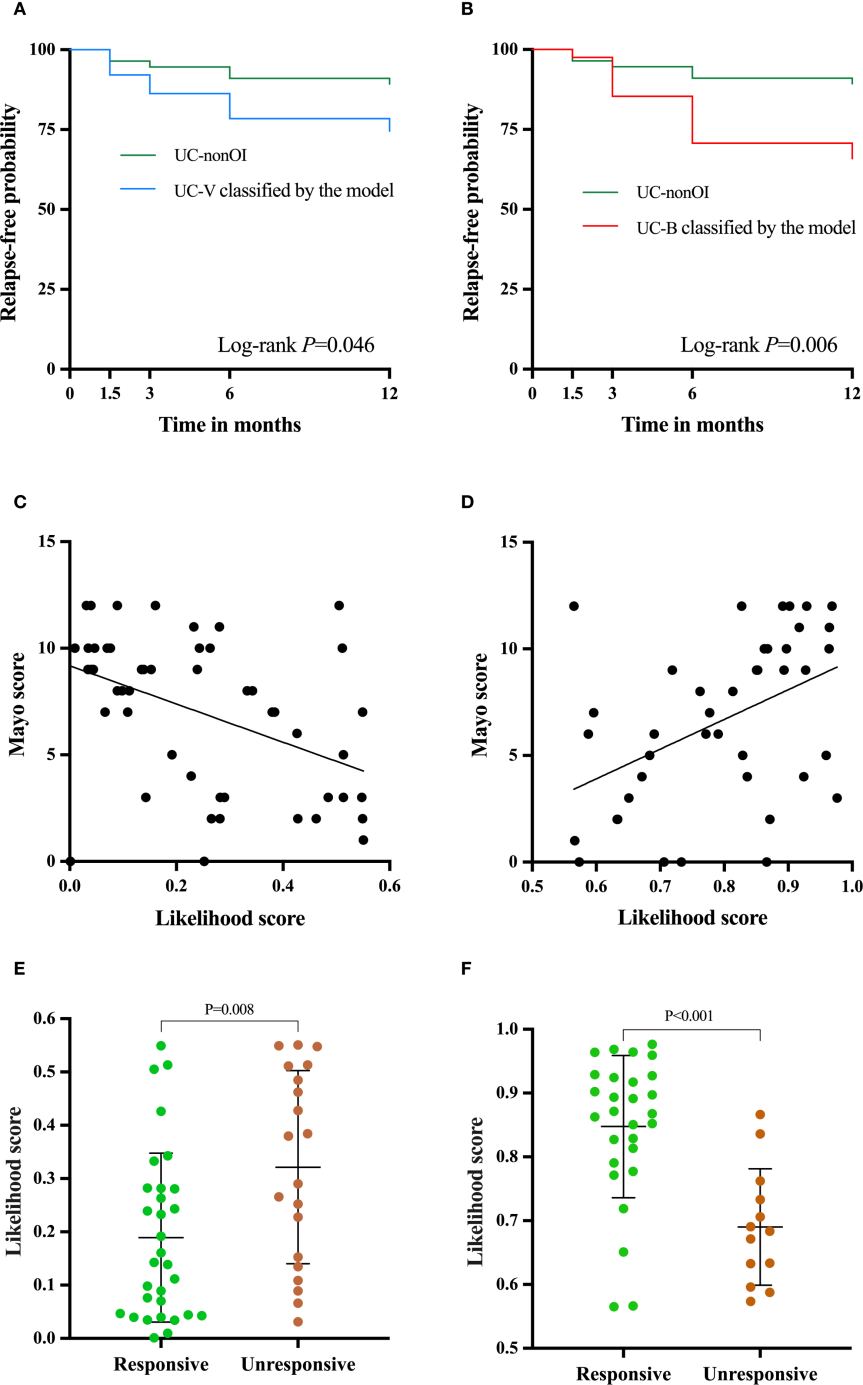
Clinical utility of the two-transcript model beyond identifying opportunistic infections. Relapse-free survival curve between UC-V **(A)** and UC-B **(B)** classified by the model and UC-nonOI group during follow-up. The correlation analysis was performed between the Likelihood scores based on the model and Mayo scores [**(C)**, UC-V; **(D)** UC-B]. Differences in the likelihood scores for response to antivirals or antibiotics [**(E)**, UC-V’s response to antivirals; **(F)**, UC-B’s response to antibiotics]. A log-rank test and independent-sample t test were used to evaluate statistical differences; Pearson’s correlation was performed to analyze the correlation.

During the follow-up of 12 months, 33 patients in validation and UC-nonOI groups (33/148, 22.3%) experienced treatment with corticosteroids, escalation to biologics or immunomodulators, hospitalization for UC-related complications and colectomy, which were proxies for disease flare. The Kaplan-Meier curve showed that the UC-V group classified by the model had a significantly higher risk of disease relapse than the UC-nonOI group (*P* = 0.046; **Figure 6A**) and the hazard ratio of the UC-V vs UC-nonOI groups for disease relapse was 2.533 (95% CI, 0.963-6.667, *P* = 0.06); the UC-B group classified by the model had a significantly higher risk of disease relapse than the UC-nonOI group (*P* = 0.006; **Figure 6B**) and the hazard ratio of the UC-B vs UC-nonOI groups for disease relapse was 3.443 (95% CI, 1.322-8.967, *P* = 0.011). Additionally, relapse risks during follow-up did not differ significantly between gold standard-defined and model-classified groups, either for UC-V (*P*>0.05; **Supplementary Figure 6A**) or UC-B (*P*>0.05; **Supplementary Figure 6B**).

### 3.9 Correlation analysis between Mayo scores and Likelihood scores based on the two-transcript model

Since the Mayo score is routinely used to evaluate the disease severity of UC, we next analyzed the correlation of Likelihood scores based on the model with Mayo scores. Interestingly, in the validation group, we observed that the likelihood score for UC-V was negatively correlated with the Mayo score (r= -0.447, *P* = 0.001; **Figure 6C**), while the likelihood score for UC-B was positively correlated with the Mayo score (r=0.454, *P* = 0.003; **Figure 6D**).

### 3.10 The two-transcript model for evaluating the efficacy of host responses to antibiotics or antivirals

In the validation group, the two-transcript model-based likelihood score for UC-V that responded to antivirals was significantly lower than in non-responders (*P* = 0.008, **Figure 6E**), whereas the score for UC-B that responded to antibiotics was significantly higher than in non-responders (*P* < 0.001, **Figure 6F**). For predicting treatment response, the model achieved an AUC of 0.708 (95% CI, 0.561-0.855; **Supplementary Figure 7A**) for antivirals in UC-V and 0.854 (95% CI, 0.734-0.975; **Supplementary Figure 7B**) for antibiotics in UC-B.

## 4 Discussion

In this study, we found that *IFI44L* expression was significantly different between UC-nonOI and UC-V and *PI3* and *ITGB2* expression demonstrated significant discrepancy between UC-nonOI and UC-B, suggesting that the transcript signature could serve as potential reference values in recognizing UC-OI. We identified the transcript levels of *IFI44L* and *PI3* differed significantly between UC-B and UC-V, which provides a new potential reference for the differential diagnosis of types of UC-OI. Moreover, the expression levels of the three candidate genes were not statistically different between HCs and UC-nonOI, suggesting that UC disease itself may not interfere with the expression patterns of these genes, thus further validating their specific role in identifying UC-OI.

In this study, we tracked the dynamics of the relative expression levels of *IFI44L*, *PI3* and *ITGB2* in the acute and recovery phases of UC-B or UC-V. The relative expression levels of *IFI44L* were higher in the acute phase of UC-B compared to the recovery phase, whereas the relative expression levels of *PI3* and *ITGB2* were not statistically different between these two clinical stages. The three candidate genes showed opposite expression trends in UC-B. These dynamic changes not only reveal the association between the expression levels of the above genes and the infection status, but also provide a potential predictor for assessing the course of infection and therapeutic efficacy in patients with UC-OI.

We established and validated a two-transcript classifier model to differentiate bacterial from viral infection in UC-OI. This model performed better than the traditional biomarkers PCT, CRP and ESR, and its performance was not affected by pathogen subtypes, showing higher stability and wider applicability. In clinical practice, trading some specificity to increase sensitivity to detect bacterial infections in patients with UC should be considered, especially in UC patients with severe and critical conditions, in whom early use of antibiotics can significantly improve prognosis and survival. However, the performance of model in distinguishing UC-OI from UC-nonOI diminishes. The primary reason for this lies in the partial overlap between the UC-V host gene expression profile and that of UC-nonOI. Specifically, many UC-V patients exhibit IFI44L expression levels comparable to those of UC-nonOI patients (both higher than UC-B but lower than healthy controls), resulting in their likelihood scores falling within an intermediate range that complicates distinction from UC-nonOI.

The two transcripts used in our model are associated with infection and the cellular origins of them provide a mechanistic basis for their discriminatory power. *IF144L*, classified within the type I interferon-induced gene group, is primarily induced in plasmacytoid dendritic cells (pDCs) and monocytes upon viral recognition, serving as a reliable biomarker for antiviral immune responses (20). *PI3* is predominantly secreted by neutrophils activated during bacterial infection, responsible for producing two distinct classes of protease inhibitors that participate in immune system modulation and the initiation of antimicrobial defense mechanisms (13, 21, 22). Although severe viral infection may cause *IFI44L* downregulation, the two-transcript model incorporating *PI3* effectively resolves this confounding factor to ensure diagnostic accuracy. Based on extensive literature review, our research appears to be the pioneering effort in employing quantitative RT-PCR analysis of these two transcripts for bacterial-viral differentiation in UC-OI.

The global challenge of antibiotic overuse persists as a critical public health concern, extending even to nations with rigorous antimicrobial stewardship programs and stringent regulatory frameworks (23, 24). This widespread issue stems from multifaceted factors, including diagnostic uncertainties, empirical treatment practices, and patient expectations. Current epidemiological data reveal that inappropriate antibiotic utilization contributes significantly to the emergence of antimicrobial resistance, creating substantial burdens on healthcare systems worldwide (24, 25). The implementation of rapid and accurate diagnostic strategies for bacterial infections has emerged as a crucial intervention point in clinical settings. Antibiotic use can have several negative effects on the gut microbiota, including reduced species diversity, altered metabolic activity, and the selection of antibiotic-resistant organisms, which in turn can lead to antibiotic-associated diarrhea and recurrent Clostridioides difficile infections (26). Therefore, in UC-OI, the use of faster and easier techniques to identify the presence or absence of bacterial infections is important for later treatment.

The previous studies of infection-related biomarkers mainly included two categories: leukocyte-associated surface proteins (CD64 and CD35) and soluble inflammatory mediators (CRP, PCT and cytokines); however, these molecular indicators demonstrate limited specificity in differentiating bacterial from viral infection (27, 28). In this study, we used RT-PCR to measure mRNA, requiring less than 5 hours from blood sampling to results. Previous research has shown that the results from different PCR platforms were highly consistent (13), suggesting that the transcript classifier can be implemented using existing PCR instrumentation in medical centers. We used PAXgene Blood RNA Tubes to collect whole blood, whose RNA stabilization reagents may improve RNA quality, further increasing the reliability of the experimental data. Although the combination of PCT and CRP is cheaper, its sensitivity and specificity are lower. Comparative analysis reveals that transcript quantification through RT-PCR demonstrates superior accessibility and economic feasibility when contrasted with next-generation sequencing (NGS), microarray technology or RNA sequencing approaches. With the emergence of novel nucleic acid amplification techniques such as microfluidic and isothermal techniques, our model could be adapted to achieve point-of-care measurement and ensure prompt administration of appropriate therapeutics (29–31).

Regarding the clinical utility of the model beyond diagnosis, our study demonstrates that the model-classified UC-V and UC-B groups exhibited significantly higher risks of disease relapse compared to UC-nonOI defined by the gold standard, and elevated hazard ratios. These findings suggest that the model identifies patients with adverse clinical outcomes who may benefit from closer monitoring or earlier intervention. Notably, relapse risks did not differ between gold standard-defined and model-classified groups, supporting the model’s reliability in replicating conventional classifications while providing additional mechanistic insights. However, there were no statistically significant differences in neutrophil counts (*P* = 0.674), ESR (*P* = 0.333), CRP (*P* = 0.890), and PCT (*P* = 0.509) between patients with recurrence (including treatment with corticosteroids, escalation to biologics or immunomodulators, hospitalization for UC-related complications and colectomy) and those without recurrence. The significant correlation between the model-based likelihood scores from UC-V and UC-B and Mayo scores demonstrates the clinical utility of the model in assessing disease activity in UC. Critically, the model predicted treatment response with moderate to high accuracy. The lower likelihood scores in antiviral responders (*P* = 0.008) and higher scores in antibiotic responders suggest that these UC-V and UC-B represent actionable therapeutic endotypes. The results support the implementation of personalized treatment strategies, which could optimize antimicrobial stewardship in UC-OI by facilitating targeted antiviral or antibiotic therapy while minimizing unnecessary antibiotic use.

Our study had several limitations. The exclusion of some UC patients due to factors such as no definitive diagnosis, suboptimal RNA samples or failed RT-PCR could introduce selection bias to some extent. Additionally, recruiting UC patients from a single tertiary medical institution may introduce bias due to the single-site sample. Future multi-center validation is needed to confirm its universality. Although our two-transcript classifier model effectively distinguishes between bacterial and viral infections in UC patients, comprehensive pathogen identification necessitates the integration of additional diagnostic modalities, including microbial cultivation and nucleic acid-based detection methods. Furthermore, *IFI44L* and *PI3* are currently supported as correlative biomarkers rather than mechanistically validated targets.

In conclusion, the two-transcript classifier model exhibited a good discrimination power, which was better than procalcitonin, CRP and ESR for discriminating bacterial from viral infection in UC-OI. The dynamic change in two transcripts expression can potentially be used to predict treatment effectiveness. These results indicate that the model is an efficient, convenient and objective differential diagnostic tool with wide potential for clinical application. Furthermore, mechanistic studies, including functional validation of candidate genes and pathway analyses, may not only elucidate novel anti-infective targets but also solidify the biological rationale underlying this model’s efficacy, representing a critical direction for future research.

## Data Availability

The raw data supporting the conclusions of this article will be made available by the authors, without undue reservation.

## Supplementary material

Supplementary Figure 1 Diagnostic process for UC with bacterial or viral infections or without opportunistic infections. * When common skin-colonized bacteria such as coagulase negative Staphylococcus were detected, the positive pathogen must be detected at least two times or in blood drew from two different sites of the body.; # Increased cerebrospinal fluid polymorphonuclear cell counts, positive cerebrospinal fluid cultures and/or positive blood cultures; @ Increased cerebrospinal fluid mononucleated cells, normal blood glucose and positive viral nucleic acids.

Supplementary Figure 2 Bioinformatics analysis. **(A)** Volcano plot of DEG analysis in GSE277849. **(B)** Volcano Plot of DEG Analysis in GSE40396. **(C)** Volcano Plot of DEG Analysis in GSE72829. **(D)** Cross-Validation Plot of LASSO Regression. DEG, differential expressed genes. LASSO, Least Absolute Shrinkage and Selection Operator.

Supplementary Figure 3 Cross-validation performance of the model. Boxplots represent the distribution of metrics across folds. The central line indicates the median, the boxes span the interquartile range (IQR), and whiskers show the 1.5×IQR range.

Supplementary Figure 4 The performance of the two-transcript model to distinguish UC-OI from UC-nonOI. The grey dashed line denotes the likelihood score value of 0.5552794, above which the UC with UC-OI is favored. PCT, CRP and ESR are included for comparison. The DeLong test was used to assess statistical differences between paired AUCs. AUC, area under the receiver operating characteristic curve; UC-B, UC with bacterial infections; PCT, Procalcitonin; CRP, C-reactive protein; ESR, erythrocyte sedimentation rate.

Supplementary Figure 5
**(A)** The likelihood scores and ROC curve based on the two-transcript model for patients with UC with EBV and bacterial infections in the validation group. **(B)** The likelihood scores and ROC curve based on the two-transcript model for patients with UC with non-EBV and bacterial infections in the validation group. The grey dashed line denotes the likelihood score value of 0.5552794, above which the UC with bacterial infections is favored. AUC, area under the receiver operating characteristic curve; UC-B, UC with bacterial infections.

Supplementary Figure 6 Relapse-free survival curve between UC-V **(A)** classified by the model and UC-V defined by the gold standard during follow-up; Relapse-free survival curve between UC-B **(B)** classified by the model and UC-B defined by the gold standard during follow-up.

Supplementary Figure 7 The receiver operating characteristic curves for model prediction of treatment response. **(A)** UC-V response to antiviral; **(B)** UC-B response to antibiotics. UC-V, UC with viral infections; UC-B, UC with bacterial infections; AUC, area under the receiver operating characteristic curve.
