# Supplementary figures and images for: A two-transcript classifier model of host genes for discrimination of bacterial from viral infection in ulcerative colitis with opportunistic infections: a discovery and validation study
Source: Front Immunol. 2025 Sep 19;16:1642923. doi: 10.3389/fimmu.2025.1642923 (PMC12491250; doi:10.3389/fimmu.2025.1642923)

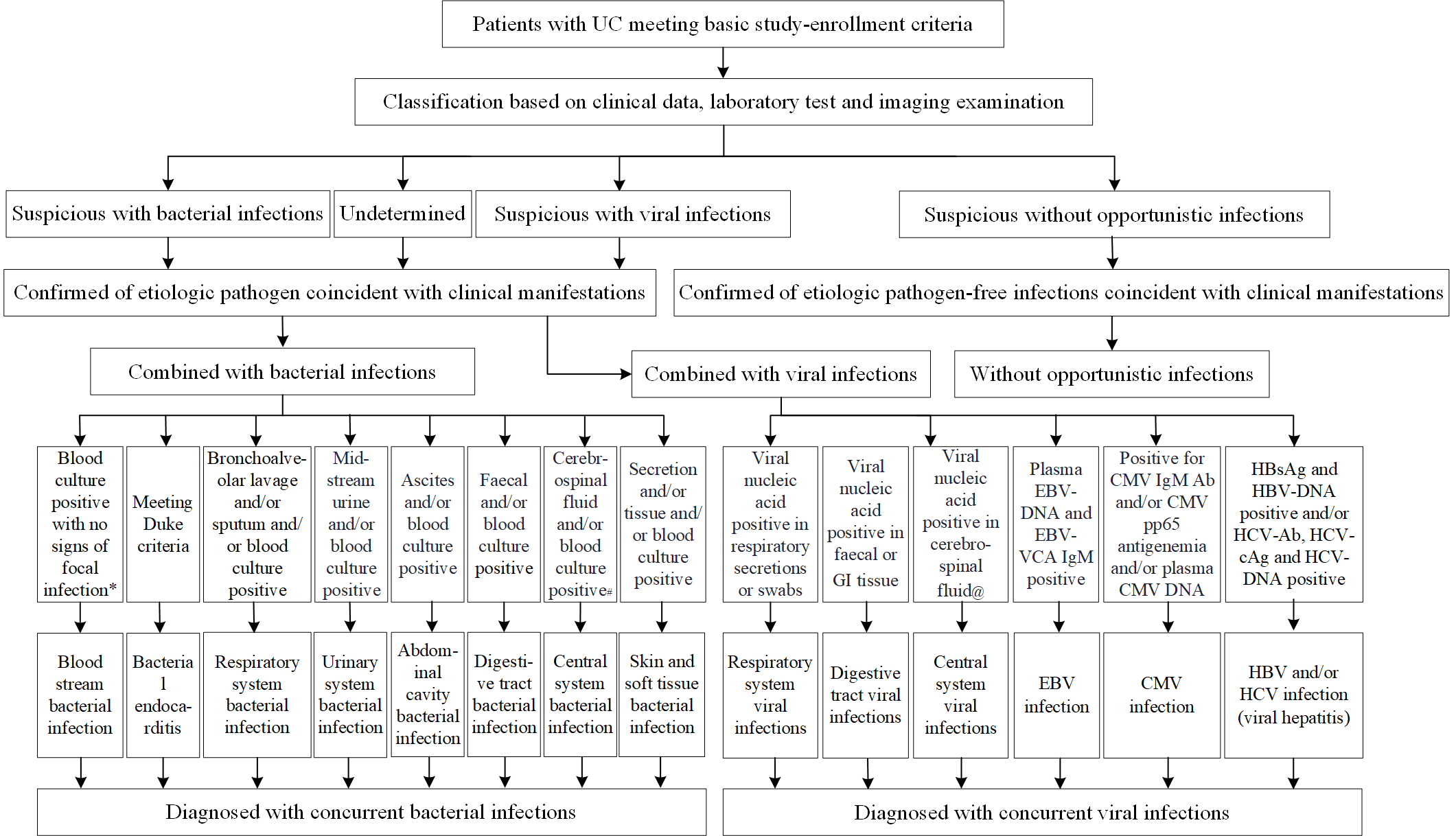

Supplement: Supplementary Figure 1 — Diagnostic process for UC with bacterial or viral infections or without opportunistic infections. * When common skin-colonized bacteria such as coagulase negative Staphylococcus were detected, the positive pathogen must be detected at least two times or in blood drew from two different sites of the body.; # Increased cerebrospinal fluid polymorphonuclear cell counts, positive cerebrospinal fluid cultures and/or positive blood cultures; @ Increased cerebrospinal fluid mononucleated cells, normal blood glucose and positive viral nucleic acids. [file Image1.tif]

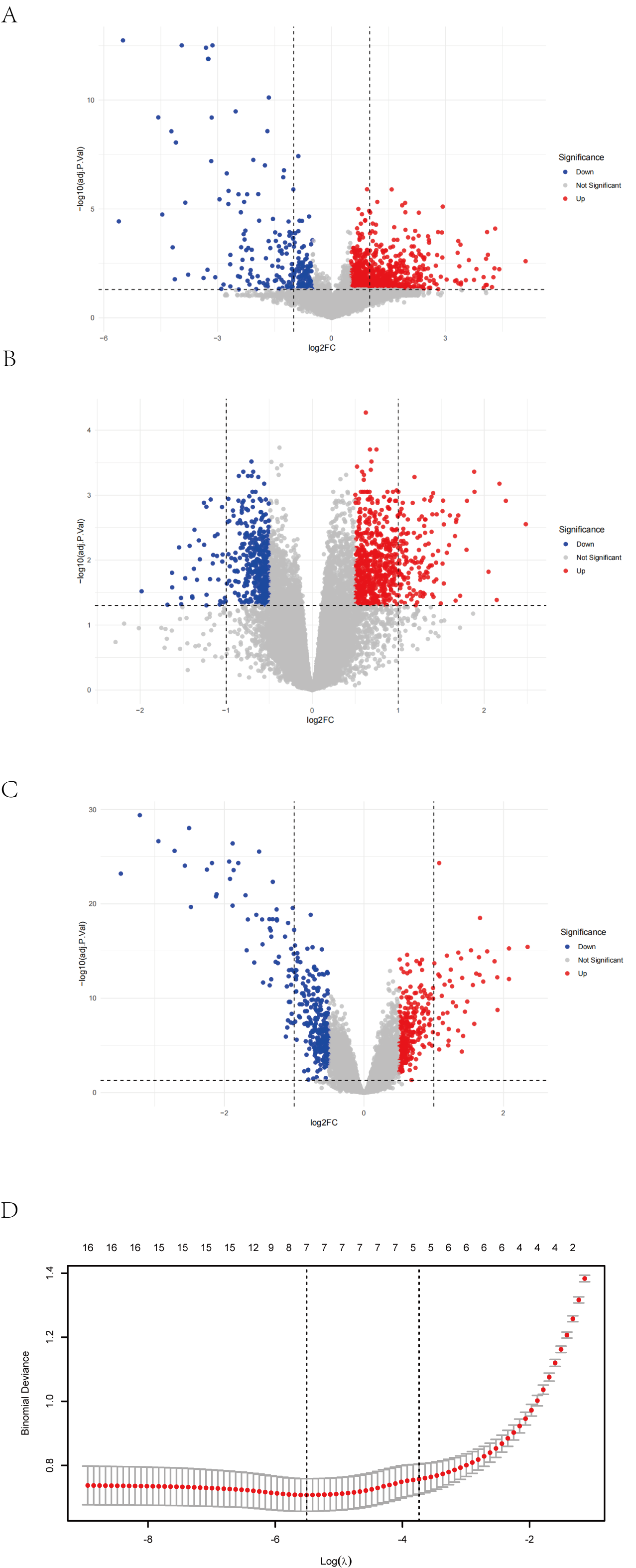

Supplement: Supplementary Figure 2 — Bioinformatics analysis. (A) Volcano plot of DEG analysis in GSE277849. (B) Volcano Plot of DEG Analysis in GSE40396. (C) Volcano Plot of DEG Analysis in GSE72829. (D) Cross-Validation Plot of LASSO Regression. DEG, differential expressed genes. LASSO, Least Absolute Shrinkage and Selection Operator. [file Image2.tif]

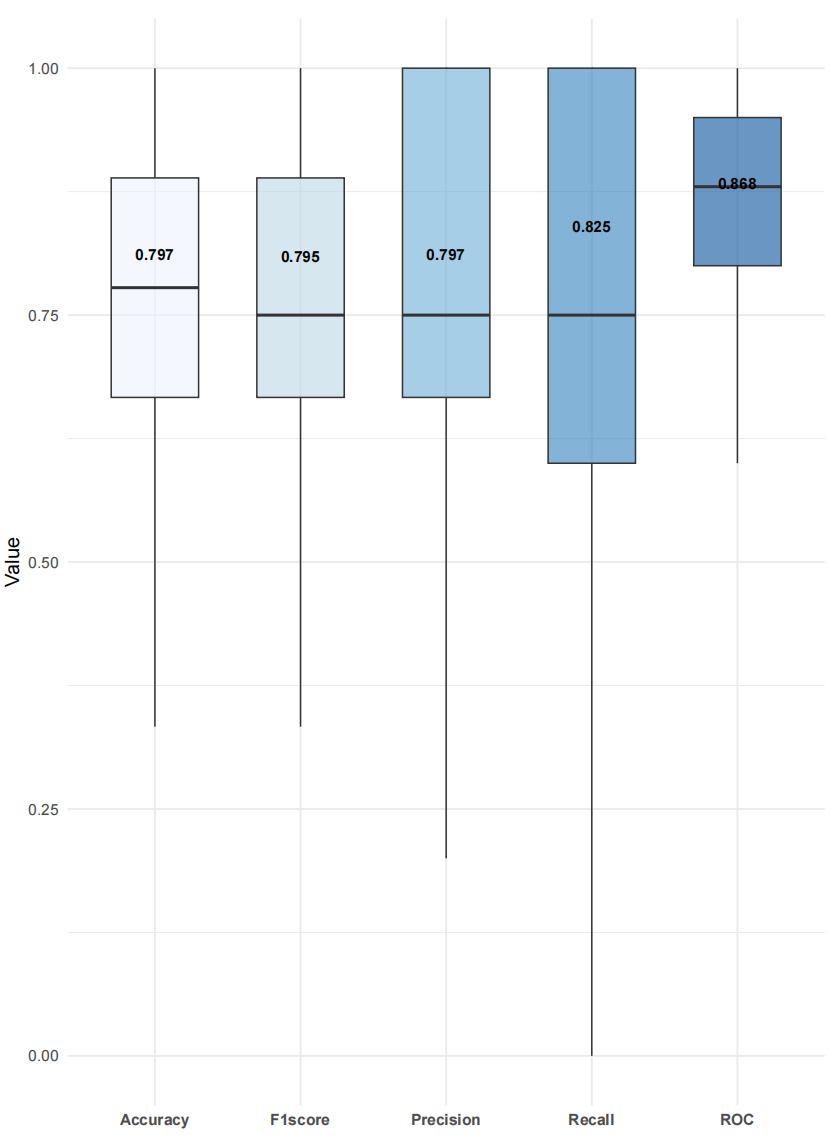

Supplement: Supplementary Figure 3 — Cross-validation performance of the model. Boxplots represent the distribution of metrics across folds. The central line indicates the median, the boxes span the interquartile range (IQR), and whiskers show the 1.5×IQR range. [file Image3.tiff]

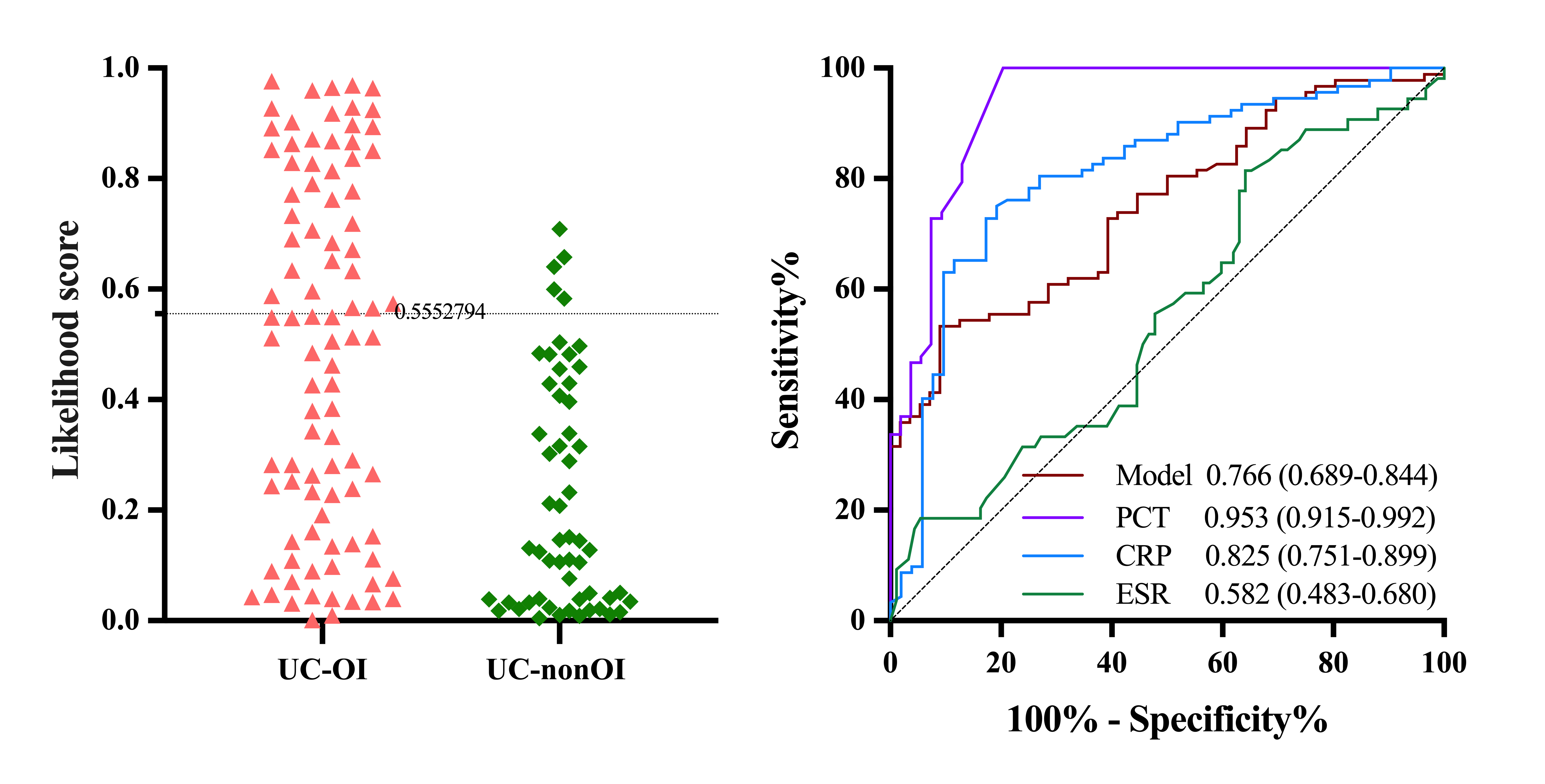

Supplement: Supplementary Figure 4 — The performance of the two-transcript model to distinguish UC-OI from UC-nonOI. The grey dashed line denotes the likelihood score value of 0.5552794, above which the UC with UC-OI is favored. PCT, CRP and ESR are included for comparison. The DeLong test was used to assess statistical differences between paired AUCs. AUC, area under the receiver operating characteristic curve; UC-B, UC with bacterial infections; PCT, Procalcitonin; CRP, C-reactive protein; ESR, erythrocyte sedimentation rate. [file Image4.tiff]

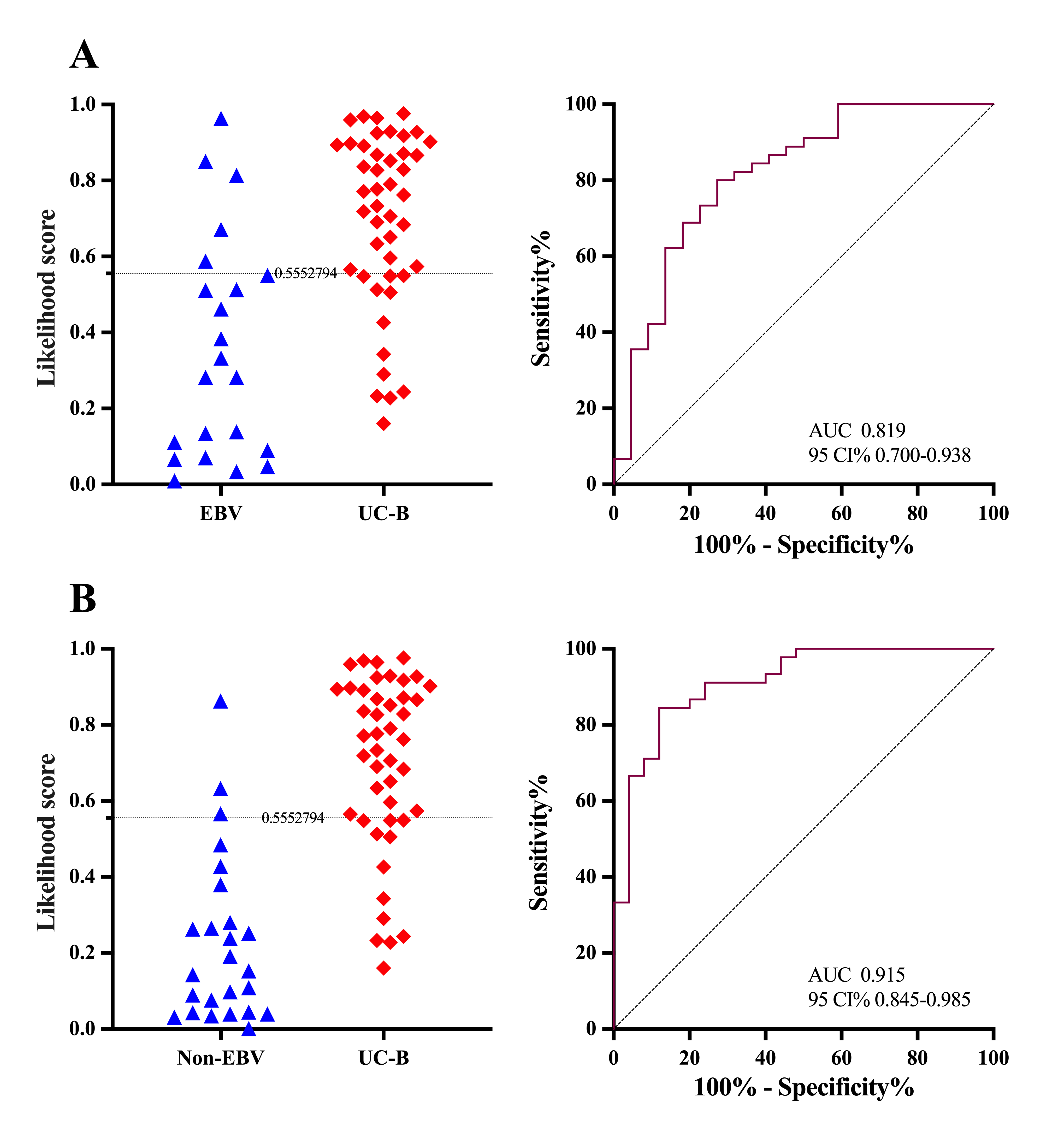

Supplement: Supplementary Figure 5 — (A) The likelihood scores and ROC curve based on the two-transcript model for patients with UC with EBV and bacterial infections in the validation group. (B) The likelihood scores and ROC curve based on the two-transcript model for patients with UC with non-EBV and bacterial infections in the validation group. The grey dashed line denotes the likelihood score value of 0.5552794, above which the UC with bacterial infections is favored. AUC, area under the receiver operating characteristic curve; UC-B, UC with bacterial infections. [file Image5.tiff]

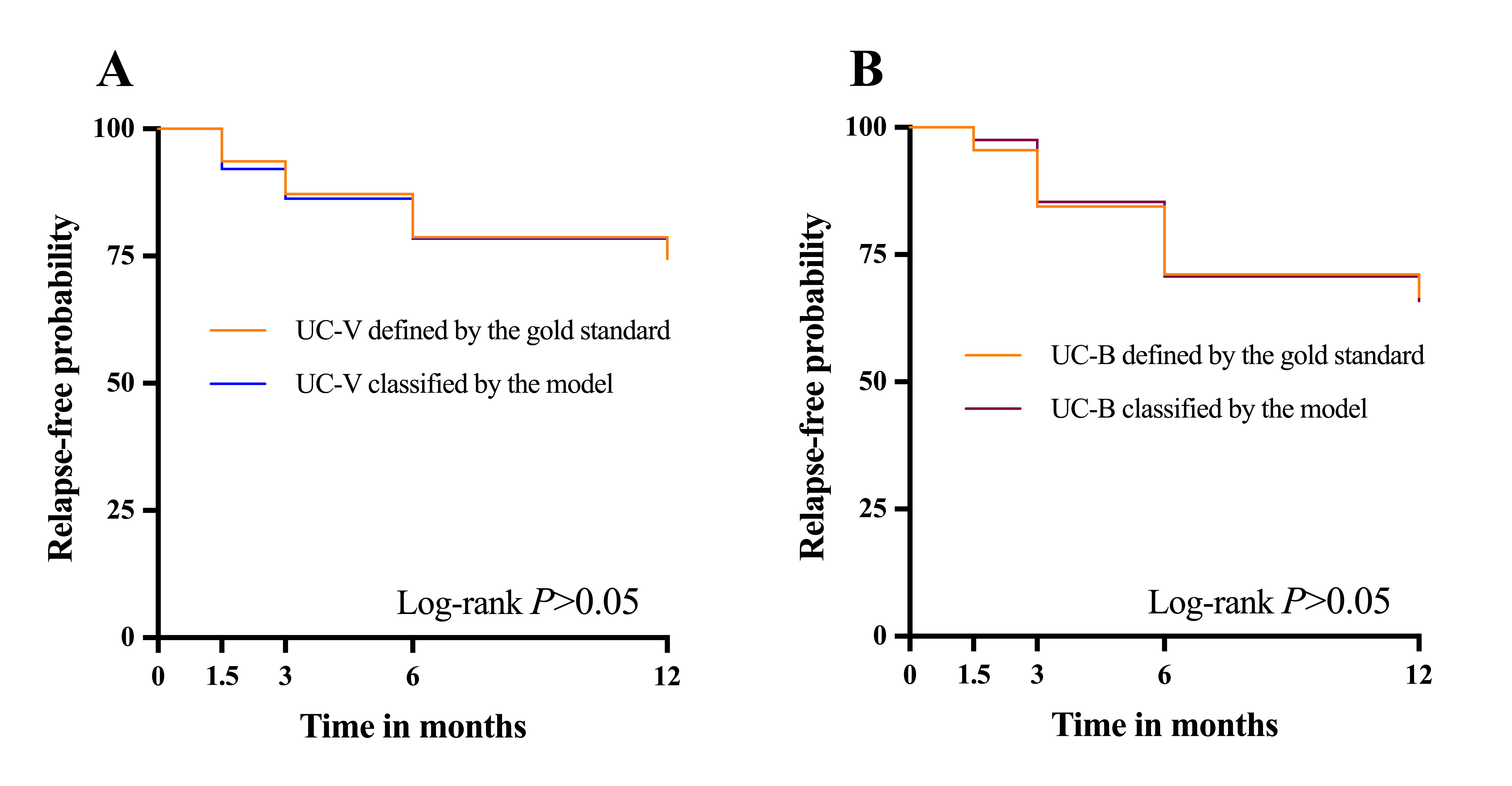

Supplement: Supplementary Figure 6 — Relapse-free survival curve between UC-V (A) classified by the model and UC-V defined by the gold standard during follow-up; Relapse-free survival curve between UC-B (B) classified by the model and UC-B defined by the gold standard during follow-up. [file Image6.tiff]

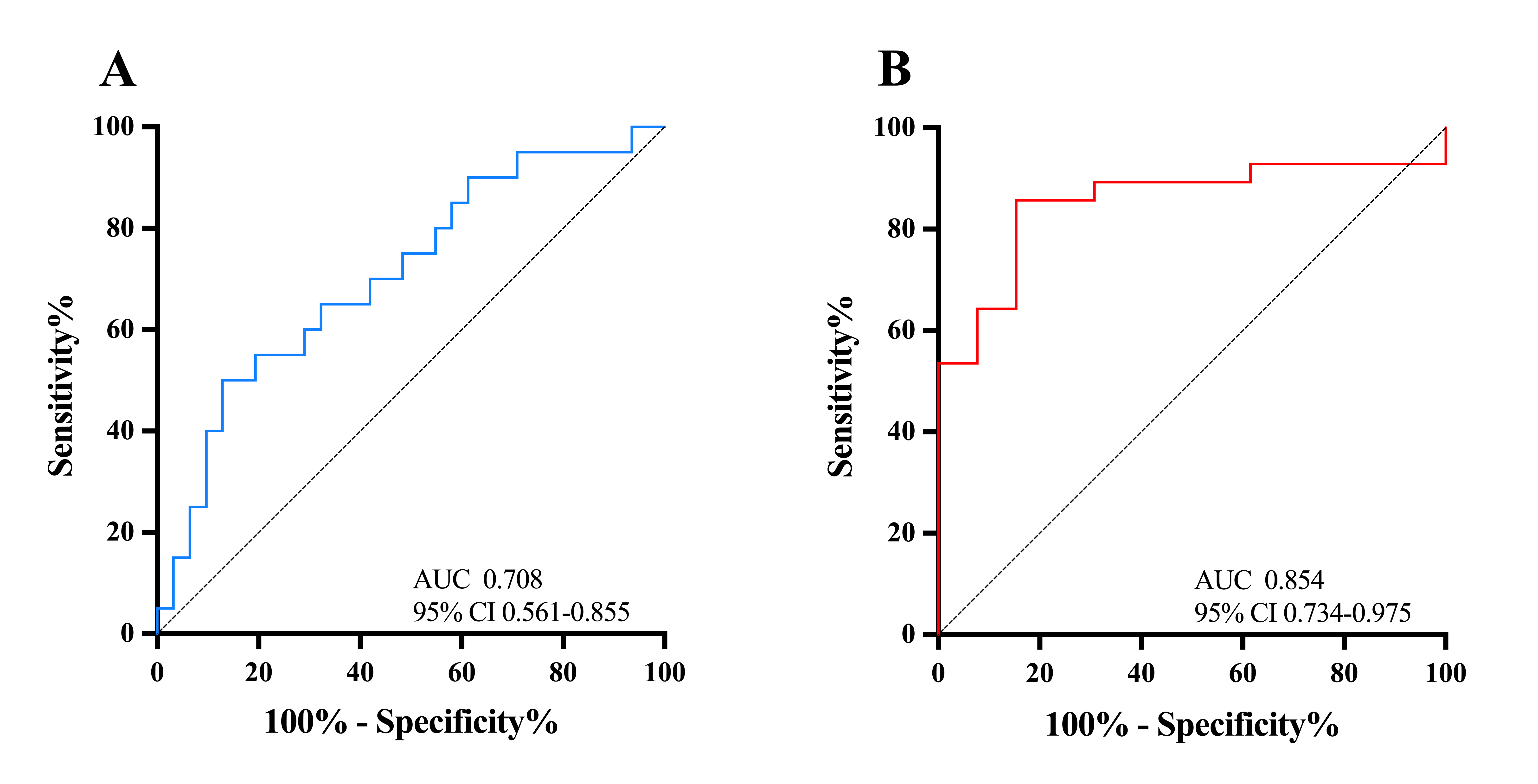

Supplement: Supplementary Figure 7 — The receiver operating characteristic curves for model prediction of treatment response. (A) UC-V response to antiviral; (B) UC-B response to antibiotics. UC-V, UC with viral infections; UC-B, UC with bacterial infections; AUC, area under the receiver operating characteristic curve. [file Image7.tiff]
