# Supplementary material for: A two-transcript classifier model of host genes for discrimination of bacterial from viral infection in ulcerative colitis with opportunistic infections: a discovery and validation study
Source: Front Immunol. 2025 Sep 19;16:1642923. doi: 10.3389/fimmu.2025.1642923 (PMC12491250; doi:10.3389/fimmu.2025.1642923)
Supplement: Supplementary file 8 [file DataSheet1.docx]

**Inclusion and exclusion criteria for UC patients**

Patients with UC included in this study were ≥14 years of age and fulfilled the diagnostic criteria for UC with confirmed bacterial or viral infections or without opportunistic infections. Confirmed combined bacterial infection was defined as a specimen from a sterile site with positive bacterial culture and/or NGS results and a clinical presentation consistent with bacterial infection. If contamination was suspected, positive results were confirmed by bacterial cultures from different sites and samples. Confirmed viral infection was defined as detection of virus by molecular techniques (PCR and/or NGS) and clinical presentation consistent with viral infection. Exclusion criteria were age <14 years, non-bacterial or viral infections, mixed infections and unclear diagnosis of infection, comorbidities that may affect host gene expression (e.g., advanced malignancy, immunodeficiency, or other systemic inflammatory diseases), pregnancy and incomplete clinical information.

**The criteria for defining the acute phase and recovery phase of opportunistic infections**

**Definition of the acute phase:**

Clinical criteria: Presence of clear symptoms of opportunistic infection (e.g., fever, worsening diarrhoea, increased mucus-blood-containing stools) and meeting any of the following laboratory evidence:

UC-B: Positive blood/stool culture or PCR detection of bacterial pathogen nucleic acid, and significantly elevated serum markers (PCT/CRP) (>2 times the upper limit of normal); UC-V: PCR detection of viral nucleic acid in blood/tissue (e.g., EBV, CMV). Timing: Blood samples collected within ≤72 hours of symptom onset.

**Definition of recovery phase:**

Clinical criteria (all of the following conditions must be met):

Complete resolution of infection symptoms (normal body temperature for ≥48 hours, diarrhoea frequency reduced to baseline levels); negative pathogen detection (negative bacterial culture or viral load below the detection limit); Inflammatory markers (PCT/CRP) return to baseline or normal range.

Timing: Collect paired blood samples 7-14 days after discontinuing antimicrobial/antiviral medications.

**Blood sample collection, processing and RT-PCR**

Blood sample collection and processing: All participants, after fasting for 8-12 hours, had blood samples collected using PAXgene Blood RNA Tubes (Becton, Dickinson and Company, USA), followed by collection in EDTA anticoagulant tubes. The samples in PAXgene Blood RNA Tubes were allowed to stand at room temperature for 2 hours before being transferred to -80°C for storage. Total RNA was extracted according to the instructions provided with the PAXgene Blood RNA Kit (Cat. No. 762174, Qiagen GmbH, Germany). Blood samples in EDTA anticoagulant tubes were stored at 4°C within 2 hours of collection for the measurement of PCT, CRP and ESR levels. PCT levels were measured using an ELISA kit (Roche Diagnostics, Brahms, Henningsdorf, Germany); CRP levels were determined by turbidimetry using the BC-5390CRP fully automated biochemical analyzer (Mindray Biomedical Electronics Co., Ltd., China); ESR levels were measured using the Westergren method, with 3.8% sodium citrate used as the anticoagulant.

RNA extraction and reverse transcription: Total RNA was extracted from the samples in PAXgene Blood RNA Tubes using the PAXgene Blood RNA Kit (Cat. No. 762174, Qiagen GmbH, Germany) within 2 hours. The purity and concentration of the extracted RNA were assessed using a NanoDrop 1000 spectrophotometer (Thermo Fisher Scientific, USA). RNA with an A260/A280 ratio greater than 1.6 was considered suitable for further analysis. The reverse transcription reaction system (20 µL) consisted of: 300 ng RNA, 1 µL random primer p(dN)6 (50 µM), 4 µL 5× reverse transcription buffer, 0.5 µL RNase inhibitor (40 U/µL), 2 µL dNTP mixture (10 mM), 1 µL M-MuLV RT (200 U/µL), and RNase-free ddH2O to a final volume of 20 µL. Reverse transcription was performed using a reverse transcription kit (Cat. No. B532435, Sangon Biotech (Shanghai) Co., Ltd., China) strictly following the manufacturer’s instructions. The resulting cDNA products were stored at -80°C for subsequent analysis.

RT-PCR: TaqMan PCR kits and gene-specific primers, including *IFI44L* (Hs00915292_m1), *PI3* (Hs00160066_m1), *ITGB2* (Hs00164957_m1), and the reference gene *ACTB* (Hs01060665_g1), were purchased from Thermo Fisher Scientific. The reaction system (20 µL) consisted of: 10 µL TaqMan Fast qPCR Master Mix, 1 µL TaqMan gene expression assay, 4 µL cDNA template, and RNase-free water to a final volume of 20 µL. Amplification was performed on a Hongshi SLAN96P real-time fluorescence quantitative PCR instrument (Hongshi Medical Technology Co., Ltd., China). A single-gene, single-channel, duplicate amplification mode was used. The PCR cycling conditions were as follows: 50°C for 2 minutes, 95°C for 10 minutes, followed by 36 cycles of 95°C for 15 seconds and 60°C for 1 minute. All samples were run in duplicate, and the average values were used for analysis. The expression levels of *IFI44L*, *PI3*, and *ITGB2* genes in each sample were normalized to the expression level of the *ACTB* gene. The relative mRNA expression levels of target genes were calculated using the δ Ct method [26], where δ Ct = (Ct value of the target gene - Ct value of the reference gene). Ct was defined as the PCR cycle number at which the fluorescence signal reached the detection threshold. The Ct values of the target and reference genes used for calculation were the average results of duplicate PCR measurements. The normalized δ Ct values were used for subsequent statistical analysis and comparison.

**Tiered Quality Control Standards for RNA Extraction, Reverse Transcription, and qPCR Workflows:**

RNA Quality Pre-screening: All RNA samples must exhibit an RNA Integrity Number (RIN) ≥ 7.0 (assessed using Agilent 2100 Bioanalyzer) and an A260/A280 ratio between 1.8 and 2.1 (though >1.6 is mentioned in the aforementioned protocol, a stricter 1.8-2.1 range is applied in practice). Samples failing to meet these criteria are excluded during the extraction stage.

Ct Value Cutoff for Internal Reference Gene: *ACTB* (β-actin) is selected as the internal reference gene. Any sample exhibiting an *ACTB* Ct value > 28 is deemed a failed sample, indicating insufficient RNA quantity or suboptimal reverse transcription efficiency. Such samples are excluded from subsequent analysis.

Target gene amplification validity check: Although Ct values for target genes (*IFI44L*, *PI3*, *ITGB2*) vary with expression levels and are not the primary criterion for sample acceptability, we implemented amplification validity quality control: All genes must exhibit a typical S-shaped amplification curve; melting curves must be unimodal to ensure amplification product specificity. Any samples exhibiting non-specific amplification or amplification failure (typically manifested as Ct values ≥ 36 or undetectable Ct values) were excluded.

**The assessment protocols for the Mayo score**

|  | **Score 0** | **Score 1** | **Score 2** | **Score 3** |
| --- | --- | --- | --- | --- |
| **Bowel movement** | Normal | 1–2 more bowel movements a day | 3–4 more bowel movements a day | ≥5 more bowel movements a day |
| **Blood in stool percentage** | No blood | Streaks of blood with stools appearing in <50% of the bowel movements | Bloody stools in >50% of the bowel movements | Frank blood mostly |
| **Physicians’ global assessments** | Normal | Mild | Moderate | Severe |
| **Endoscopic findings** | Normal | Inflammation | Erosion | Ulcer |

The Mayo score measures disease severity by integrating clinical symptoms, such as bowel movement (score 0-3; symptoms ranging from normal, 1-2 more bowel movements a day, 3-4 more bowel movements a day or ≥5 more bowel movements a day), blood in stool percentage (score 0–3; no blood, streaks of blood with stools appearing in <50% of the bowel movements, bloody stools in >50% of the bowel movements or frank blood mostly), physicians’ global assessments (score 0-3) and endoscopic findings (score 0-3; normal, inflammation, erosion or ulcer). The total Mayo score ranges from 0 to 12, with remission having a score of 0-2 (no subscores >1), mild disease activity having a score of 3-5, moderate to severe disease activity having a score of 6-10, and severe disease activity having a score of 11-12.
