# Supplementary material for: A two-transcript classifier model of host genes for discrimination of bacterial from viral infection in ulcerative colitis with opportunistic infections: a discovery and validation study
Source: Front Immunol. 2025 Sep 19;16:1642923. doi: 10.3389/fimmu.2025.1642923 (PMC12491250; doi:10.3389/fimmu.2025.1642923)
Supplement: Supplementary file 9 [file Table1.docx]

**Supplementary Table 1.** Demographic and clinical characteristics of the study participants

|  | **UC-V [n=77]** | **UC-B [n=75]** | **P-Value^#^** | **UC [n=56]** | **HC [n=72]** | **P-Value^*^** |
| --- | --- | --- | --- | --- | --- | --- |
| **Age (years)** | 50.39±13.202 | 47.05±13.749 | 0.129 | 45.39±14.610 | 48.85±12.186 | 0.147 |
| **Male, n [%]** | 46 [59.74%] | 47 [62.67%] | 0.711 | 34 [60.71%] | 42 [58.33%] | 0.786 |
| **Duration of disease (years)** | 3 [1.5, 10] | 2.5 [0.9, 10] | 0.138 | 3 [1, 10] |  |  |
| **Neutrophil count [10^9/L]** | 4.922±2.159 | 7.265±3.787 | <0.001 | 3.896±1.366 | 4.012±1.449 | 0.648 |
| **Lymphocyte count [10^9/L]** | 1.843±0.691 | 1.735±0.701 | 0.341 | 1.689±0.584 | 1.640±0.588 | 0.643 |
| **Monocyte count [10^9/L]** | 0.581±0.641 | 0.481±0.212 | 0.200 | 0.381±0.162 | 0.386±0.169 | 0.842 |
| **Albumin level (mg/dL) ^a^** | 37.45±5.424 | 38.40±6.054 | 0.311 | 41.57±6.766 | 44.81±4.113 | <0.001 |
| **Hemoglobin level (g/L)** | 111.05±25.002 | 113.48±25.706 | 0.556 | 120.87±27.676 | 144.50±12.515 | <0.001 |
| **Platelet count [10^9/L]** | 313.9±127.46 | 336.2±132.29 | 0.294 | 304.19±109.93 | 282.56±67.99 | 0.206 |
| **ESR (mm/h) ^b^** | 24.0 [13.3, 51.5] | 33.0 [20.0, 65.0] | 0.019 | 28.0 [12.5, 44.0] | 14.0 [8.0, 18.0] | <0.001 |
| **CRP level (mg/dL) ^c^** | 18.3 [8.8, 45.8] | 47.8 [30.4, 81.0] | <0.001 | 3.5 [1.1, 9.4] | 0.6 [0.3, 4.5] | <0.001 |
| **Procalcitonin (ng/mL) ^d^** | 0.20 [0.08, 0.48] | 0.84 [0.20, 3.46] | <0.001 | 0.04 [0.02, 0.07] | 0.02 [0.02, 0.02] | <0.001 |

Data are presented as mean ± standard deviation or median (P_25_, P_75_) or count (percentage). Statistical differences were assessed using independent samples t-test, Fisher's exact test, and Mann-Whitney U test. ^a^ Data on albumin levels were missing for 5 study participants; ^b^ data on ESR levels were missing for 3 study participants; ^c^ data on CRP levels were missing for 4 study participants; ^d^ Data on PCT levels were missing for 2 study participants. ^#^ *P*-value for comparison between UC-V and UC-B groups; ^*^ *P*-value for comparison between UC and HC groups. UC-V: UC with viral infections, UC-B: UC with bacterial infections, UC: UC without opportunistic infections, ESR: sedimentation, CRP: C-reactive protein, PCT: procalcitonin.

**Supplementary Table 2.** Relative expression levels of candidate genes of the study participants in four subgroups

|  | **UC-V (n=77)** | **UC-B (n=75)** | **UC (n=56)** | **HC (n=72)** |
| --- | --- | --- | --- | --- |
| ***IFI44L*** | 8.954±2.594 | 11.808±2.073 | 12.186±3.791 | 12.902±3.577 |
| ***PI3*** | 9.697±3.472 | 6.946±2.461 | 11.564±4.077 | 12.270±4.299 |
| ***ITGB2*** | 3.621±0.732 | 3.401±0.620 | 3.830±0.875 | 4.056±0.931 |

The relative expression level of candidate genes was expressed as δ Ct values, and higher δ Ct values indicate lower mRNA expression. Data are presented as mean ± standard deviation. UC-V, UC with viral infections; UC-B, UC with bacterial infections; UC, UC without opportunistic infections; HCs, healthy controls.

**Supplementary Table 3.** The diagnostic performance of pairwise combination of *IFI44L*, *PI3*, and *ITGB2* in the discovery group by logistic regression.

|  | **AUC** | **95%CI** |
| --- | --- | --- |
| ***IFI44L* and *PI3*** | 0.862 | 0.760-0.964 |
| ***IFI44L* and *ITGB2*** | 0.832 | 0.726-0.938 |
| ***PI3* and *ITGB2*** | 0.768 | 0.645-0.891 |

**Supplementary Table 4.** Multivariate Logistic regression analyses of UC with bacterial infections

|  | **B** | **S.E** | **OR** | **95% CI** | ***P*-Value** |
| --- | --- | --- | --- | --- | --- |
| ***IFI44L*** | 0.579 | 0.166 | 1.785 | 1.289-2.470 | <0.001 |
| ***PI3*** | -0.444 | 0.146 | 0.641 | 0.482-0.854 | 0.002 |
| ***ITGB2*** | -1.433 | 0.669 | 0.239 | 0.064-0.886 | 0.032 |

Abbreviations: S.E, Standard Error.
